# An Exploration of Psychiatry Residents’ Morbidity and Mortality Education Through a Structured Curriculum to Teach Self-Reflection, Accountability, and Systems-Based Learning

**DOI:** 10.1007/s40596-026-02307-x

**Published:** 2026-01-28

**Authors:** Angela Le, Cassandra Haworth, Cory Nielsen, Kathleen Mathieson, Claire Sollars

**Affiliations:** Creighton University School of Medicine - Phoenix, Phoenix, AZ USA

**Keywords:** Curriculum, Morbidity and mortality, Resident education

## Abstract

**Objective:**

This study describes a psychiatry residency curriculum integrating root cause analysis (RCA), reflective practice, and systems-based thinking within morbidity and mortality (M&M) conferences.

**Methods:**

As part of post-graduate year 2 (PGY-2) didactics, 40 residents and recent graduates (*n* = 40) completed a 9-week RCA course culminating in a formal, resident-led M&M conference from 2018 to 2023. A one-time anonymous electronic survey was distributed to past participants in March 2024. The survey included 20 Likert scale items assessing knowledge, skills, and attitudes aligned with Accreditation Council for Graduate Medical Education (ACGME) competencies.

**Results:**

Respondents (*n* = 29; 100%) agreed or strongly agreed with 13 of 20 survey items, particularly regarding understanding of systems issues and contributing factors in adverse events. Twenty-nine respondents (100%) reported the course fostered respect and psychological safety. Items receiving neutral or disagree responses primarily reflected self-assessments of ability to challenge judgments or discuss their mistakes.

**Conclusions:**

A structured curriculum combining reflective practice with objective RCA effectively enhanced psychiatry residents’ knowledge, skills, and attitudes across multiple ACGME competencies. Although limited by single site-design, modest sample size, and recall bias, this model represents a scalable approach to teaching patient safety and systems-based practice in psychiatry training.

The Accreditation Council for Graduate Medical Education (ACGME) requires residents to participate in quality improvement and analysis of patient safety events, as well as practice-based learning and improvement as part of their core competencies [1]. Morbidity and mortality (M&M) conferences are well established in surgical and medical training; however, their structure and implementation vary [2–5]. Prior work has noted both the relative absence of psychiatry-specific M&M models and the potential value of resident-driven approaches [6]. Common goals of the M&M conference include fostering meaningful practice improvement, improving evidence-based practice, and increasing residents’ comfort and confidence in analyzing safety issues [7, 8]. A key element in achieving these goals is creating an environment of professionalism, confidentiality, and psychological safety early in training. Despite its importance, there is limited literature detailing a curriculum designed to impart and explore these reflective principles while analyzing adverse events from a systems-based perspective [9–12]. Existing models often emphasize either didactic case presentations or root cause analysis, but rarely combine the two in a structured, longitudinal framework.

To address this gap, we developed a curriculum that explicitly aligns with all six ACGME core competencies while introducing a novel dual approach: first engaging residents in reflective analysis of cases they were personally involved in, and then transitioning to objective, systems-based review of cases outside their direct care. This structure not only reinforces psychological safety and accountability but also cultivates a comprehensive, competency-driven learning experience. By integrating these dimensions, the curriculum seeks to strengthen residents’ competence across all six ACGME core competencies, as detailed below.

1. Patient Care: Residents analyze adverse events to identify contributing factors and propose changes that improve patient safety and quality of care.
2. Medical Knowledge: Residents apply foundational knowledge of psychiatry, patient safety, and RCA methods, integrating evidence-based guidelines into case analysis.
3. Systems-Based Practice: Residents identify systemic contributors to adverse events using the fishbone diagram, propose quality improvement (QI) initiatives, and connect learning to institutional processes.
4. Practice-Based Learning and Improvement: Structured reflection on personal cases helps residents evaluate their own practice, recognize biases, and incorporate feedback into future care.
5. Interpersonal and Communication Skills: Residents interview staff (when appropriate), lead peer discussions, and present cases to a multidisciplinary audience, fostering clear and respectful communication.
6. Professionalism: The course emphasizes confidentiality, respect, and accountability, cultivating psychological safety and reducing blame culture.

## Methods

The post-graduate year 2 (PGY-2) course, Morbidity and Mortality Conference: A Systems-based Approach, has been required since 2017 for general psychiatry residents at a single academic program. As of 2023, 48 residents had completed the course. For this study, eligible participants included all graduates and current residents who completed the course between 2018 and 2023 (*n* = 40). Contact information for the initial cohort in 2017 was unavailable.

The Part 1 nine-week didactic course is introduced early in PGY-2 and consists of 60-min sessions, which are held consecutively to provide a cohesive foundational introduction to morbidity and mortality principles. Instruction blends brief didactics with discussion-based methods (dialogic inquiry, Socratic questioning, and case-based learning) to promote active learning and psychological safety. Part 2, consisting of larger M&M sessions, was distributed throughout the remainder of the academic year to allow scheduling flexibility, facilitate participation from additional faculty members, and provide residents with opportunities to apply and reinforce course concepts in clinical practice between sessions. The course director, a faculty psychiatrist, serves as a mentor throughout the process, facilitating reflection, modeling non-punitive language, and ensuring confidentiality. The curriculum is intentionally divided into two parts: Part 1: Didactic and Reflective Learning and Part 2: Objective, Systems-Based Analysis.

In Part 1, residents first learn foundational principles of patient safety, root cause analysis, and morbidity and mortality through course director assigned readings and guided discussions that emphasize patient safety, clinician education, transparency, and collaboration [13–16]. Articles highlight implementation challenges in psychiatry training, such as undefined adverse outcomes, blame culture, and lack of structured forums. There are also articles to discuss areas of improvement, such as diagnostic errors, communication breakdowns, systems issues, and cultural misunderstandings. Residents learn how to use the modified Ishikawa fishbone diagram, a tool originally developed as a cause-and-effect diagram for QI initiatives, to systematically evaluate adverse events and identify systems-level improvements [17]. During the final five weeks, in facilitated small-group discussions, each resident analyzes and informally presents a case involving an adverse event in which they were personally involved to fellow classmates to cultivate self-awareness, accountability, and bias recognition. Class analysis then ensues, using the modified Ishikawa fishbone diagram to identify systemic factors.

In Part 2, the curriculum culminates in a formal department-wide, resident-led M&M presentation on a case in which they did not provide direct care (e.g., identified through routine clinical exposure, such as while on call). This shift allows for an objective, systems-level investigation of an adverse event, free from the emotional complexity of personal involvement. Before any chart review, Department Chair approval is required per institutional policy. Presentations rely on de-identified information only; no protected health information is disclosed. There are a variety of topics that may be discussed, including (but not limited to) unexpected death, major medical complications, extended length of stay, early readmission, delays in initiating treatment or care, suicide attempts, self-destructive or self-harming behavior, violence, staff injury, adverse medication reactions, prolonged seclusion and restraint (S&R) events, incorrect medication or dose administered, incorrect or missed diagnosis, repeated emergency room transfers, or elopements. Once approval is obtained, residents may review the medical record, relevant guidelines and policies, and, when appropriate, interview involved team members to identify systems-based contributors, such as communication breakdowns or workflow vulnerabilities. At the conclusion of the formal presentation, residents submit potential quality improvement (QI) ideas to psychiatry leadership for further consideration.

In March 2024, a one-time, anonymous electronic survey was distributed via REDCap by an independent third party to all eligible participants who had completed the course between 2018 and 2023 (*n* = 40)[18]. The survey instrument, developed for this curriculum evaluation, is available from the corresponding author upon request. The instrument contained 20 Likert scale items (1 = Strongly Disagree to 5 = Strongly Agree) and one optional free-response item, developed from prior reflective practice and resident-led M&M curricula [19, 20]. The survey was reviewed for content and face validity by the research team and external fellows.

Before starting the survey, all participants reviewed an electronic information sheet and provided informed consent by clicking “Yes” or “I Consent”; those who declined were redirected to a termination page. This included PGY-2 residents from the classes of 2024–2027 and attending physicians who completed residency between 2018 and 2023. To assess the impact of the curriculum, the survey included items evaluating participants’ knowledge, skills, and attitudes, as described below:

### Knowledge

Understanding the benefits and components of root cause analysis and systems-based practice through the use of a modified Ishikawa fishbone diagram as a framework for analyzing adverse events. This approach involves examining systems-based factors, including medical knowledge, diagnostic reasoning, therapeutic choices, clinical assessment, materials/machines, personnel, communication, processes, and environmental influences that contributed to the adverse event.

### Skills

Applying the fishbone diagram as a framework for root cause analysis to investigate patient safety events from a systems perspective, focusing on systemic factors rather than individual blame, while fostering reflective practice, accountability, and conscientious decision-making.

### Attitudes

Developing comfort and confidence in root cause analysis, fostering commitment to self-reflection, accountability, well-being, and psychological safety.

Data was imported into IBM SPSS Statistics for analysis. Frequencies and percentages were reported for all survey items. Analysis was limited to post-course self-assessment as no pre-course measures were collected. The Institutional Review Board determined the project was not human subjects research and therefore exempt from review. All case materials and presentations were de-identified.

## Results

We evaluated the curriculum’s success by assessing improvements in knowledge, skills, and attitudes related to root cause analysis, systems-based practice, reflective practice, accountability, well-being, and psychological safety, aligning with ACGME competencies. A total of 40 surveys were distributed, and 30 (75%) were returned. One participant was excluded because they did not check the consent box, resulting in 29 (73%) responses used in analysis. Fifty-five percent of participants identified as female, and 45% identified as male. Participants included 17 residents (55%) and 12 attendings (45%).

Survey results indicated strong perceived benefits across all target ACGME core competencies (Table 1). The majority of respondents agreed or strongly agreed with all survey items. Across the 20 items, no more than 13.8% (*n* = 4) of respondents selected neutral, disagree, or strongly disagree response options for any single item. For 13 of the 20 items, 100% (*n* = 29) of respondents selected *agree* or *strongly agree.* Items with the highest *strongly agree* responses included: better understanding of systems issues related to adverse events, understanding contributing factors that impact patient safety, reviewing factors that contribute to adverse events, and everyone was treated with respect throughout the course.

**Table 1:** Responses to survey items

**Survey items**	Strongly disagree	Disagree	Neutral	Agree	Strongly agree
	*n* (%)	*n* (%)	*n* (%)	*n* (%)	*n* (%)
This course helped me better understand systems issues as they are related to adverse events	0	0	0	8 (27.6)	21 (72.4)
This course was a valuable part of my education	0	0	0	9 (31.0)	20 (69.0)
This course has helped me feel more confident in my ability to identify and discuss errors from a systems-based perspective	0	0	0	11 (37.9)	18 (62.1)
The course has helped me better understand the contributing factors that impact patient safety	0	0	0	8 (27.6)	21 (72.4)
This course provided the skills for reviewing factors that contribute to adverse events	0	0	0	8 (27.6)	21 (72.4)
Think about how the system has influenced and impacted my own involvement in patient care	0	0	0	15 (51.7)	14 (48.3)
Consider other alternatives and factors that may impact patient care	0	0	1 (3.4)	12 (41.4)	16 (55.2)
Intentionally reflect on my own involvement and peers' involvement in cases with openness and humility	0	0	0	12 (41.4)	17 (58.6)
Realize the importance of life-long reflection and commitment to personal growth	0	0	3 (10.3)	7 (24.1)	19 (65.5)
Challenge my own judgements and beliefs	0	2 (6.9)	2 (6.9)	9 (31.0)	16 (55.2)
Hold myself accountable	0	0	1 (3.4)	16 (55.2)	12 (41.4)
Take ownership of the role that I played in outcomes	0	0	0	15 (51.7)	14 (48.3)
See an experience from different perspectives	0	0	0	11 (37.9)	18 (62.1)
Be more aware of my own limitations	0	0	2 (6.9)	11 (37.9)	16 (55.2)
Discuss adverse events objectively without shame and blame	0	0	0	13 (44.8)	16 (55.2)
After this course, going forward, I would be more willing to discuss my own mistakes	0	0	3 (10.3)	15 (51.7)	11 (37.9)
This course helped promote an environment that was non-threatening	0	0	1 (3.4)	10 (34.5)	18 (62.1)
Everyone was treated with respect throughout the course	0	0	0	7 (24.1)	22 (75.9)
This course helped me debrief and learn from other adverse outcomes	0	0	0	11 (37.9)	18 (62.1)

Only one item received any level of disagreement: 6.9% (*n* = 2) of respondents selected *disagree* with the statement, “This course helped me challenge my own judgments and beliefs.” All other items with neutral responses reflected participants’ perceptions of their own abilities or the impact of the course, including self-accountability, the importance of lifelong reflection, awareness of personal limitations, and willingness to discuss their own mistakes as well as considering alternatives that impact patient care and promoting an environment that is non-threatening. No respondent selected *strongly disagree.* The data set, modified Ishikawa fishbone diagram, and free response are available upon contacting the corresponding author.

## Discussion

Morbidity and Mortality (M&M) conferences are essential in residency training, yet there is limited literature on how to effectively teach underlying systems-based and humanistic principles. Residents and faculty believed this curriculum resulted in benefits across multiple ACGME competencies, enhancing residents’ knowledge, skills, and attitudes toward patient safety, root cause analysis, psychological safety, self-reflection, accountability, and lifelong learning. Despite a small sample size from a single institution, findings suggest that the curriculum strengthens these foundational competencies and has a meaningful impact on training.

A distinguishing feature of this curriculum lies in its design: one that strategically supports both introspective learning and objective, systems-based evaluation of clinical events. During the implementation weeks, each resident selects and presents a clinical case in which they were directly involved. This personal involvement facilitates emotionally engaged, reflective discussions grounded in lived experience. By analyzing how health systems impact their own clinical decision-making, residents deepen their self-awareness, develop accountability, and gain insight into the complex factors that contribute to adverse events. These peer-facilitated discussions create a supportive, nonjudgmental space to explore these events through a learning lens—fostering trust, psychological safety, and collective learning.

For the final presentation, residents select a case they were not personally involved in, using only the medical record for analysis. This shift, from emotionally involved to emotionally uninvolved, allows for objective, systems-based analysis without the emotional complexity of personal involvement. The chart review process allows residents to analyze systems issues, communication breakdowns, or workflow vulnerabilities that may not be apparent in real time.

Survey responses provided valuable data about participants’ experiences, with all respondents affirming that the course was a meaningful part of their education. Notably, among the seven survey items receiving neutral or disagree responses, four pertained to self-perceived abilities. Participants’ confidence in factual knowledge contrasted with cautious self-assessments of ability, likely reflecting greater awareness of the complexity and demands of applying what they learned.

While results are promising, limitations must be acknowledged. The sample size was small and at one training program, which limits generalizability. Participant recall bias may have influenced the accuracy of feedback, as several respondents were recent graduates reporting retrospectively. Attending physicians, despite their greater clinical experience, are possibly subject to recall bias, as they base their view of the M&M process on the time elapsed since their training. Response bias should also be taken into account; respondents with strong opinions (either positive or negative) may have been more likely to complete the survey. Social desirability bias could also play a role, with respondents possibly overreporting the benefits of the curriculum. Pre- and post-intervention assessments were not obtained, preventing conclusions about objective changes in competency development over time. To mitigate potential biases and strengthen the evidence base, future research should employ objective pre- and post-outcome measures and engage larger, multi-institutional, and demographically diverse cohorts to more fully evaluate the curriculum’s effectiveness and generalizability.

By shifting M&M discussions away from individual blame and toward collaborative, systems-based problem-solving, this curriculum fosters professionalism, empathy, and respect. It cultivates self-awareness, clinical reasoning, and social belonging while reducing burnout and promoting reflective practice.

This innovative curriculum not only enhances residents’ ability to analyze adverse events but also fosters a culture of psychological safety essential to constructive discussion and lifelong learning. Its success provides a scalable model for psychiatry and other interdisciplinary healthcare training programs seeking to reshape how adverse event analysis is taught. With the curriculum’s focus on reflective practice, accountability, and a systems-based approach to patient safety, its broader dissemination and further development have the potential to redefine the teaching of adverse event analysis in medical education.

## Data Availability

Full data set available upon emailing the corresponding author.
